# Cellulose dissolution in diallylimidazolium methoxyacetate + *N*-methylpyrrolidinone mixture

**DOI:** 10.1038/s41598-019-48066-8

**Published:** 2019-08-08

**Authors:** Airong Xu, Yongxin Wang, Rukuan Liu

**Affiliations:** 1 School of Chemical Engineering and Pharmaceutics, Henan University of Science and Technology, Luoyang, Henan 471003 P.R. China; 2 Hunan Academy of Forestry, Changsha, Hunan 410004 P.R. China

**Keywords:** Materials chemistry, Process chemistry

## Abstract

The utilization of cellulose in industrial applicat is of great significance to sustainable development of human society and reducing dependence on dwindling fossil resources. Nevertheless, this utilization of cellulose has actually been limited due to its insolubilization. Here, novel solvents consisting of diallylimidazolium methoxy acetate ([A_2_im][CH_3_OCH_2_COO]) and *N-*methylpyrrolidinone (NMP) were developed. The solubility of cellulose in [A_2_im][CH_3_OCH_2_COO]/NMP was determined, and the influence of [A_2_im][CH_3_OCH_2_COO]/NMP molar ratio on cellulose dissolution was systematically investigated. Meanwhile, we also presented the affecting factors of the cellulose material fabrication including preparation approach, [A_2_im][CH_3_OCH_2_COO] and cellulose solution concentration. Attractively, the [A_2_im][CH_3_OCH_2_COO]/NMP solvents display much powerful dissolution capacity for cellulose even at 25 °C (25.4 g 100 g^−1^). This is mainly ascribed to the combined factors: The hydrogen bond interactions of the H2, H4 and H6 in [A_2_im]^+^ and carboxyl O atom in [CH_3_OCH_2_COO]^−^ with the hydroxyl H atom and O atom in cellulose; the dissociation of NMP towards [A_2_im][CH_3_OCH_2_COO]; the stabilization of NMP towards the dissolved cellulose chains. In addition, the thermostability and chemical structure of the regenerated cellulose from the solvents was also estimated.

## Introduction

Cellulose is a highly abundant biopolymer resource in nature with fascinating properties such as biodegradability, biocompatibility, non-toxicity, and so on^1–3^. The utilization of this resource in industrial applications is of great significance to sustainable development of human society and reducing dependence on dwindling fossil resources. Nevertheless, the extensive utilization of cellulose is actually warded off in that cellulose is extremely difficult to be dissolved owing to its enormous hydrogen bond network^4–7^. As such, many efforts have been made to develop novel and efficient solvents of cellulose. The developed solvents include *N*-methylmorpholine-*N*-oxide, lithium chloride + *N*,*N*-dimethylacetamide, tetrabutyl ammonium fluoride + dimethyl sulfoxide, NaOH/Thiourea, ionic liquids (ILs), and so on^8–13^.

Among the above solvents, the ILs receive increasing attention based on their unique properties such as negligible vapor pressure, non-flammability, high chemical and thermal stability, and strong dissolution ability for various organic and inorganic materials^14–17^. The dissolution and processing of cellulose in ILs were firstly initiated by Rogers and coworkers in 2002. They found that 10 wt% of cellulose solubility in 1-butyl-3-methylimidazolim chloride could be obtained at 100 °C, and the cellulose solubility (25 wt%) was markedly improved via microwave heating^18^. Afterward, a number of anion/cation-functionalized ILs have been developed to reduce viscosity of ILs, improve dissolution capacity for cellulose or decrease cellulose dissolution temperature. The reported ILs include 1-allyl-3-methylimidazolium chlorides^19^ alkylimidazolium carboxylates^20–26^, imidazolium dialkylphosphates^27^, choline amino acids/carboxylates^28,29^, quaternary ammonium chlorides^30^, and tetraalkylammonium carboxylate^31^. By comparison, some imidazolium carboxylate ILs displayed better dissolution performance for cellulose including 1-butyl-3-methylimidazolim acetate and propionate ILs, 1-allyl-3-methylimidazolium acetate, propionate and butyrate ILs, and 1, 3-diallylmethylimidazolium acetate, butyrate, acrylate, methoxyacetate and ethoxyacetate ILs. However, it was noted that neat ILs are generally viscous, causing the difficult dispersion of cellulose in them. Accordantly, complete cellulose dissolution generally required long residue time or a rise in temperature which can give rise to a degradation of the ILs and/or cellulose. To overcome the issues, some more efficient cellulose solvent systems composed of IL and cosolvent have recently been developed. Among them are 1-butyl-3-methylimidazolium chloride + cosolvent (dimethyl sulfoxide, *N*,*N*-dimethylformamide, etc.)^32^, 1-butyl-3-methylimidazolium acetate + cosolvent (dimethyl sulfoxide, *N*,*N*-dimethylformamide or *N*,*N*-dimethylacetamide)^33–36^, 1, 3-diallylimidazolium methoxyacetate + cosolvent (dimethyl sulfoxide, *N,N*-dimethylformamide or *N,N*-dimethylacetamide)^37^ and 1-allyl-3-methylimidazolium acetate + PEG^38^. The solvent systems display lower dissolution temperature, higher cellulose solubility and lower viscosity than neat ILs.

This work aims to develop novel solvents which were expected to more efficiently dissolve cellulose than the solvents reported previously, and fabricate the cellulose material with varying morphologic structures using the solvents. Therefore, we here design novel solvents by combining [A_2_im][CH_3_OCH_2_COO] with NMP, and the solubilities of cellulose in the solvents were determined at 25 °C. Meanwhile, the influences of NMP/[A_2_im][CH_3_OCH_2_COO] molar ratio on cellulose dissolution and the possible dissolution mechanism for cellulose were investigated. Additionally, the characterization of the regenerated cellulose from [A_2_im][CH_3_OCH_2_COO]/NMP solvent was completed to examine its chemical structure and thermostability.

## Materials and Methods

### Materials

Microcrystalline cellulose (MCC) was purchased from Sigma Aldrich Company, its viscosity-average degree of polymerization is 270. *N*-Allylimidazole (99%), allyl chloride (98%), ethoxyacetic acid (98%) and anion exchange resin (Ambersep 900-OH) were purchased from Alfa Aesar Company. *N*-Methylpyrrolidinone (NMP) (>99.9%) was purchased from Shanghai Aladdin biochemical technology Co., Ltd., and dried using 4 A molecular sieve before use. [A_2_im][CH_3_OCH_2_COO] was synthesized according to the method reported previously^20^.

### Cellulose solubility determination and preparation of porous cellulose materials PCM-1, PCM-3, PCM-5, and PCM-7

The determination of cellulose solubility in [A_2_im][CH_3_OCH_2_COO]/NMP solvents is similar to the procedures described in the literatures^35^. Typically, 1.8 g of [A_2_im][CH_3_OCH_2_COO]/NMP(*R*_NMP_ = 0.50) solvent was added to a 20 mL of glass flask, where *R*_NMP_ represented the molar ratio of NMP to A_2_im][CH_3_OCH_2_COO]. Then, the flask was immersed in a oil bath (25 ± 0.5 °C). Cellulose (0.1 g per 100 g solvent) was added to the solvent. The [A_2_im][CH_3_OCH_2_COO]/NMP(*R*_NMP_ = 0.50)/cellulose mixture was stirred until cellulose was thoroughly solubilized. Then, additional cellulose was added. The procedure was repeated until cellulose could not be solubilized. The thorough dissolution of cellulose was monitored by a polarization microscope. If cellulose was thoroughly dissolved, the mixture was optically clear under the polarization microscope. Cellulose solubility at 25 °C was calculated based on the amount of solvent and cellulose added. The cellulose solubility data at 25 °C were shown in Table 1.

**Table 1:** The solubility (g 100 g^−1^) of cellulose in [A_2_im][CH_3_OCH_2_COO]/NMP at 25 °C.

Solvents	*R*_NMP_	Solubility
[A_2_im][CH_3_OCH_2_COO] (*R*_NMP_ = 0)	0	16.2
[A_2_im][CH_3_OCH_2_COO]/NMP (*R*_NMP_ = 0.41)	0.41	20.5
[A_2_im][CH_3_OCH_2_COO]/NMP (*R*_NMP_ = 0.81)	0.81	24.1
[A_2_im][CH_3_OCH_2_COO]/NMP (*R*_NMP_ = 1.22)	1.22	25.4
[A_2_im][CH_3_OCH_2_COO]/NMP (*R*_NMP_ = 2.43)	2.43	17.5
[A_2_im][CH_3_OCH_2_COO]/NMP (*R*_NMP_ = 4.87)	4.87	12.2
[A_2_im][CH_3_OCH_2_COO]/NMP (*R*_NMP_ = 7.30)	7.30	3.4
[A_2_im][CH_3_OCH_2_COO]/NMP (*R*_NMP_ = 14.61)	14.61	2.7
NMP(*R*_NMP_ = 1)	—^a^	0

*R*_NMP_ is the molar ratio of NMP to [A_2_im][CH_3_OCH_2_COO].

^a^*R*_NMP_ is not indicated for neat NMP.

As an example, cellulose was dissolved in [A_2_im][CH_3_OCH_2_COO]/NMP(*R*_NMP_ = 1) solvent to gain 1% of solution. The solution was poured in a Petri dish, taken off air bubble under vacuum for 30 min at ambient temperature. Then, the Petri dish containing the solution was immersed in distilled water to obtain cellulose hydrogel. The hydrogel was repeatedly washed with distilled water to remove [A_2_im][CH_3_OCH_2_COO] and NMP. The washed hydrogel was frozen for 8 h at −20 °C, and then freeze-dried in a FD-10 freeze-dryer (Henan Brother Equipment Co. Ltd., China) to obtain porous cellulose material. The material was named as PCM-1. PCM-3, PCM-5 and PCM-7 porous materials from 3%, 5% and 7% of cellulose solutions were prepared via a similar procedure to PCM-1, respectively.

### ^13^C NMR and FTIR spectra measurements

Cellulose was dissolve in [A_2_im][CH_3_OCH_2_COO]/NMP(*R*_NMP_ = 2) solvent to gain [A_2_im][CH_3_OCH_2_COO]/NMP(*R*_NMP_ = 2)/cellulose(8%) solution in which cellulose solubility is 8.0%. The ^13^C NMR spectra of [A_2_im][CH_3_OCH_2_COO] in [A_2_im][CH_3_OCH_2_COO]/NMP(*R*_NMP_ = 2) solvent and [A_2_im][CH_3_OCH_2_COO]/NMP(*R*_NMP_ = 2)/cellulose(8%) solution were measured on a Bruker DMX 300 spectrometer at room temperature. DMSO**-***d*_6_ was used as an external standard. Chemical shifts were given in ppm downfield from TMS.

Cellulose was dissolve in [A_2_im][CH_3_OCH_2_COO]/NMP(*R*_NMP_ = 2.43) solvent to gain [A_2_im][CH_3_OCH_2_COO]/NMP(*R*_NMP_ = 2)/cellulose(9.0%) solution in which cellulose solubility is 9.0%. Measurements of FTIR spectra for [A_2_im][CH_3_OCH_2_COO] in [A_2_im][CH_3_OCH_2_COO]/NMP(*R*_NMP_ = 2.43) solvent and [A_2_im][CH_3_OCH_2_COO]/NMP(*R*_NMP_ = 2.43)/cellulose solution were performed on a spectrometer Nicolet iN10 spectrometer with Ge crystal ATR accessory at room temperature. Spectra were collected in high-resolution mode (4 cm^−1^ resolution and 64 scans) under an ATR 5% maximum pressure.

## Results and Discussion

### Relationship between cellulose solubility and NMP/[A_2_im][CH_3_OCH_2_COO] molar ratio

Table 1 shows the solvents with varying molar ratio of NMP to [A_2_im][CH_3_OCH_2_COO] and the solubility data of cellulose in [A_2_im][CH_3_OCH_2_COO]/NMP solvents. Since the thermal stability of a compound is vital in view of its practical application, the thermal decomposition temperature of [A_2_im][CH_3_OCH_2_COO] was determined. Its thermal decomposition temperature is 203 °C. Moreover, NMP is a extensively used solvent which is known to be chemical and thermal stability. Therefore, the [A_2_im][CH_3_OCH_2_COO]/NMP solvents have good thermal stabilities. As can be seen, NMP/[A_2_im][CH_3_OCH_2_COO] molar ratio significantly affects cellulose solubility. The cellulose solubility increases as NMP content increases in the molar ratio range 0–1.22, reaches 25.4 g 100 g^−1^ of maximum cellulose solubility in [A_2_im][CH_3_OCH_2_COO]/NMP (*R*_NMP_ = 1.22) solvent, and then decreases in the molar ratio range from 1.22 to 14.61. More interestingly, the [A_2_im][CH_3_OCH_2_COO]/NMP (*R*_NMP_ = 0.41 – 2.43) solvents display much powerful dissolution capacity for cellulose with as high as 17.5–25.4 g 100 g^−1^ of cellulose solubilities, much higher than [C_4_mim][CH_3_COO]/DMSO, [C_4_mim][CH_3_COO]/DMF and [C_4_mim][CH_3_COO]/DMA solvents^33,34,36^. For example, the solubility of cellulose in [A_2_im][CH_3_OCH_2_COO]/NMP (*R*_NMP_ = 1.22) solvent is higher than that in [C_4_mim][CH_3_COO]/DMSO solvent by about 69% at 25 °C, which has been reported to be the most efficient cellulose solvent to date. It is also found that the solubility of cellulose in neat [A_2_im][CH_3_OCH_2_COO] is 16.2 g 100 g^−1^ at 25 °C, but cellulose cannot be dissolved in NMP at this temperature. This suggests that [A_2_im][CH_3_OCH_2_COO] in [A_2_im][CH_3_OCH_2_COO]/NMP solvent mainly contributes to the cellulose dissolution.

Polar aprotic solvents could disassociate ILs into cation and anion which readily interacted with cellulose to promote cellulose dissolution^39,40^. Therefore, after NMP (one of polar aprotic solvents) is added to [A_2_im][CH_3_OCH_2_COO], the concentrations of disassociated [A_2_im]^+^ and [CH_3_OCH_2_COO]^−^ increase as NMP content increases. Correspondingly, the cellulose solubility also increases as NMP content increases. However, the further increase of NMP content could decrease the concentrations of [A_2_im]^+^ and [CH_3_OCH_2_COO]^−^ in [A_2_im][CH_3_OCH_2_COO]/NMP solvent. Consequently, the cellulose solubility decreased with increasing NMP content behind the maximum cellulose solubility at *R*_NMP_ = 1.22.

Based on the above analysis, it can be drawn a conclusion that the addition of NMP to [A_2_im][CH_3_OCH_2_COO] considerably enhances cellulose solubility. At a proper NMP/[A_2_im][CH_3_OCH_2_COO] molar ratio range, the addition of NMP to [A_2_im][CH_3_OCH_2_COO] is apparently favorable to cellulose dissolution owing to the increased A_2_im]^+^ and [CH_3_OCH_2_COO]^−^ concentrations. However, the further NMP addition decreases A_2_im]^+^ and [CH_3_OCH_2_COO]^−^ concentrations, and thus cellulose solubility. The efficacy of NMP in [A_2_im][CH_3_OCH_2_COO]/NMP solvent is mainly to disassociate [A_2_im][CH_3_OCH_2_COO] into “free” [A_2_im]^+^ and [CH_3_OCH_2_COO]^−^ which are responsible for cellulose dissolution. At the same time, it was found that, at the same molar ratio range of [A_2_im][CH_3_OCH_2_COO] to polar aprotic solvents, the cellulose solubility decreased in the order [A_2_im][CH_3_OCH_2_COO]/NMP > [A_2_im][CH_3_OCH_2_COO]/DMSO > [A_2_im][CH_3_OCH_2_COO]/DMF > [A_2_im][CH_3_OCH_2_COO]/DMA solvents^37^, where DMSO, DMF and DMA represent dimethyl sulfoxide, *N,N*-dimethylformamide and *N*,*N*-dimethylacetamide, respectively. This indicates that the cosolvent effects the cellulose dissolution, which is consistent with the results reported previously^32–36^. In addition, it was found that absorbent cotton (viscosity-average degree of polymerization of 1586) could be dissolved in [A_2_im][CH_3_OCH_2_COO]/NMP(*R*_NMP_ = 2.43) solvent at 50 °C while it was very difficult to dissolve in conventional solvents. It was also observed that 5.2% cellulose solution is very viscous and difficult to be stirred (see Fig. S1).

### ^13^C NMR and FTIR analysis of Cellulose dissolution mechanism

**Figure 1:**
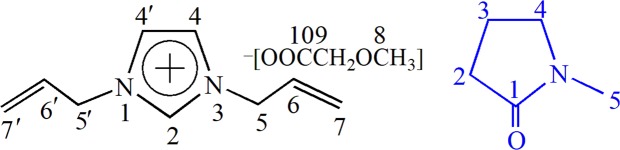
Schematic structure and serial number of [A_2_im][CH_3_OCH_2_COO] (left) and NMP (right).

To investigate the possible dissolution mechanism of cellulose in [A_2_im][CH_3_OCH_2_COO]/NMP solvent, The ^13^C NMR spectra of [A_2_im][CH_3_OCH_2_COO] in [A_2_im][CH_3_OCH_2_COO]/NMP(*R*_NMP_ = 2) solvent and [A_2_im][CH_3_OCH_2_COO]/NMP(*R*_NMP_ = 2)/cellulose(8%) solution were determined at room temperature and shown in Figs S2 and S3. The ^13^C NMR data of [A_2_im][CH_3_OCH_2_COO] were given in Table 2. Schematic structure and numbering of C atoms of [A_2_im][CH_3_OCH_2_COO] and NMP were shown in Fig. 1.

**Table 2:** The ^13^C NMR chemical shifts (*δ* (ppm) relative to TMS) of [A_2_im][CH_3_OCH_2_COO] in [A_2_im][CH_3_OCH_2_COO]/NMP(*R* = 2) solvent and in the mixture of [A_2_im][CH_3_OCH_2_COO]/NMP(*R* = 2)/cellulose(8%) solution at room temperature.

Cellulose concentration (%)	*δ* (ppm)							
	C2	C4	C5, 5′	C6, 6′	C7, 7′	C8	C9	C10
0	138.1	122.9	57.3	132.6	119.4	50.6	72.9	172.9
8	137.4	122.8	57.4	132.3	119.6	50.7	72.5	173.6
∆*δ*	−0.7	−0.1	0.09	−0.3	0.2	0.1	−0.4	0.7

It is clear that, after the addition of cellulose to [A_2_im][CH_3_OCH_2_COO]/NMP(*R*_NMP_ = 2) solvent, the signals of the C2 and C4 atoms in imidazolium ring markedly moves upfield (a marked decrease of chemical shift). This indicates that in [A_2_im][CH_3_OCH_2_COO]/NMP(*R*_NMP_ = 2)/cellulose(8%) solution, as the results of the interaction between the acidic H2, H4 protons and the hydroxyl oxygen atoms in cellulose through hydrogen bonding, the electron cloud density of the C2 and C4 atoms increased, leading to the upfield of their chemical shifts. Moreover, the hydrogen bond interaction between H2 proton and the hydroxyl oxygen in cellulose are stronger than that between H4 proton and the hydroxyl oxygen in cellulose. The carboxyl C10 atom demonstrates the signal considerably moved downfield (chemical shift increases significantly). This suggests that there’s strong hydrogen bond formed between the carboxyl oxygen atom in [CH_3_OCH_2_COO]^−^ and the hydroxyl proton of cellulose. The electron cloud density of C10 atom thus decreases to move its chemical shift downfield. Meanwhile, strong interaction between the hydroxyl oxygen in cellulose and H6 atom leads to the upfield movement of the signal of C6 atom on allyl chain. Moreover, the electron cloud density redistribution may cause the upfield shift of C9 atom and downfield shift of C7 atom. In addition, O8 interacts with hydroxyl hydrogen atom through the hydrogen bond to increase the chemical shift of C8 atom. Nevertheless, the chemical shift of C5 atom remains still. Based on analysis above, the main driving force of the cellulose dissolution in [A_2_im][CH_3_OCH_2_COO]/NMP solvent principally derives from the interactions of the H2, H4 and H6 atoms in [A_2_im]^+^ with the hydroxyl oxygen in cellulose along with the carboxyl oxygen atom in [CH_3_OCH_2_COO]^−^ with the hydroxyl hydrogen in cellulose.

Moreover, it is also to note that observable chemical shift variations (0.27 ppm) for C1 atom of NMP are observed, indicating that the carboxyl O atom in NMP can interact with the hydroxyl H atoms of dissolved cellulose molecule by forming hydrogen bonds. This interaction can further prevent the dissolved cellulose chains from reformation of their inter- and intramolecular hydrogen bonds. Additionally, the signals of C2 (0.04 ppm), C3 (0.07 ppm), C4 (0.07 ppm) and C5 (0.02 ppm) atoms remain nearly invariable, implying that the H atoms bonded to the C atoms hardly have interactions with the cellulose hydroxyls.

Taken together, the cellulose dissolution could attribute to the H2, H4 and H6 atoms in [A_2_im]^+^ and the carboxyl oxygen atom in [CH_3_OCH_2_COO]^−^. To validate this conclusion, the FTIR spectra of [A_2_im][CH_3_OCH_2_COO] in [A_2_im][CH_3_OCH_2_COO]/NMP(*R*_NMP_ = 2.43) solvent and [A_2_im][CH_3_OCH_2_COO]/NMP(*R*_NMP_ = 2.43)/cellulose(9%) solution were further elucidated under room temperature. The C-H stretching vibration of the carbon-carbon double bound (C4 = C4′, C6 = C7) is known to be weak. Moreover, the C6, 7-H stretching vibration of the C6 = C7 double bound in allyl groups and C4 = C4′ double bound in [A_2_im]^+^ is overlaped. Additionally, the strength of the C-O asymmetric stretching vibration in [CH_3_OCH_2_COO]^−^ is greater than its symmetric part. The discussion next will concentrate upon the C2-H stretching vibration of [A_2_im]^+^ and C-O stretching vibration in [CH_3_OCH_2_COO]^−^.

**Figure 2:**
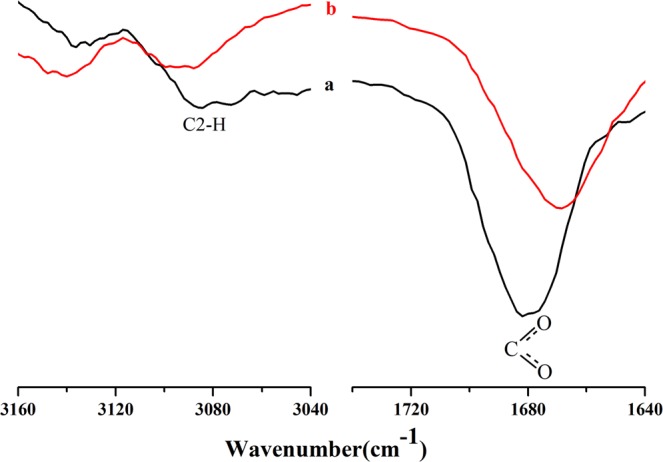
FTIR spectra of the C2-H stretching vibration in [A_2_im]^+^ and C-O stretching vibration in [CH_3_OCH_2_COO]^−^: (**a**) [A_2_im][CH_3_OCH_2_COO]/NMP(*R*_NMP_ = 2.43) solvent; (**b**) [A_2_im][CH_3_OCH_2_COO]/NMP(*R*_NMP_ = 2.43)/cellulose solution containing 9% of cellulose.

Figure 2 shows the FTIR spectra of the C2-H stretching vibration of [A_2_im]^+^ and C-O stretching vibration in [CH_3_OCH_2_COO]^−^. As shown in Fig. 2, in addition of cellulose to [A_2_im][CH_3_OCH_2_COO]/NMP(*R*_NMP_ = 2.43) solvent, the C2-H stretching at around 3091 cm^−1^ displayed blue-shift, and meanwhile the C-O stretching at around 1602 cm^−1^, exhibited red-shift. This is mainly due to the hydrogen bond interactions of the H2 atom in [A_2_im]^+^ with the hydroxyl oxygen in cellulose and the carboxyl oxygen atom in [CH_3_OCH_2_COO]^−^ with the hydroxyl hydrogen in cellulose^41^.

### Morphology and structure of the porous cellulose material

**Figure 3:**
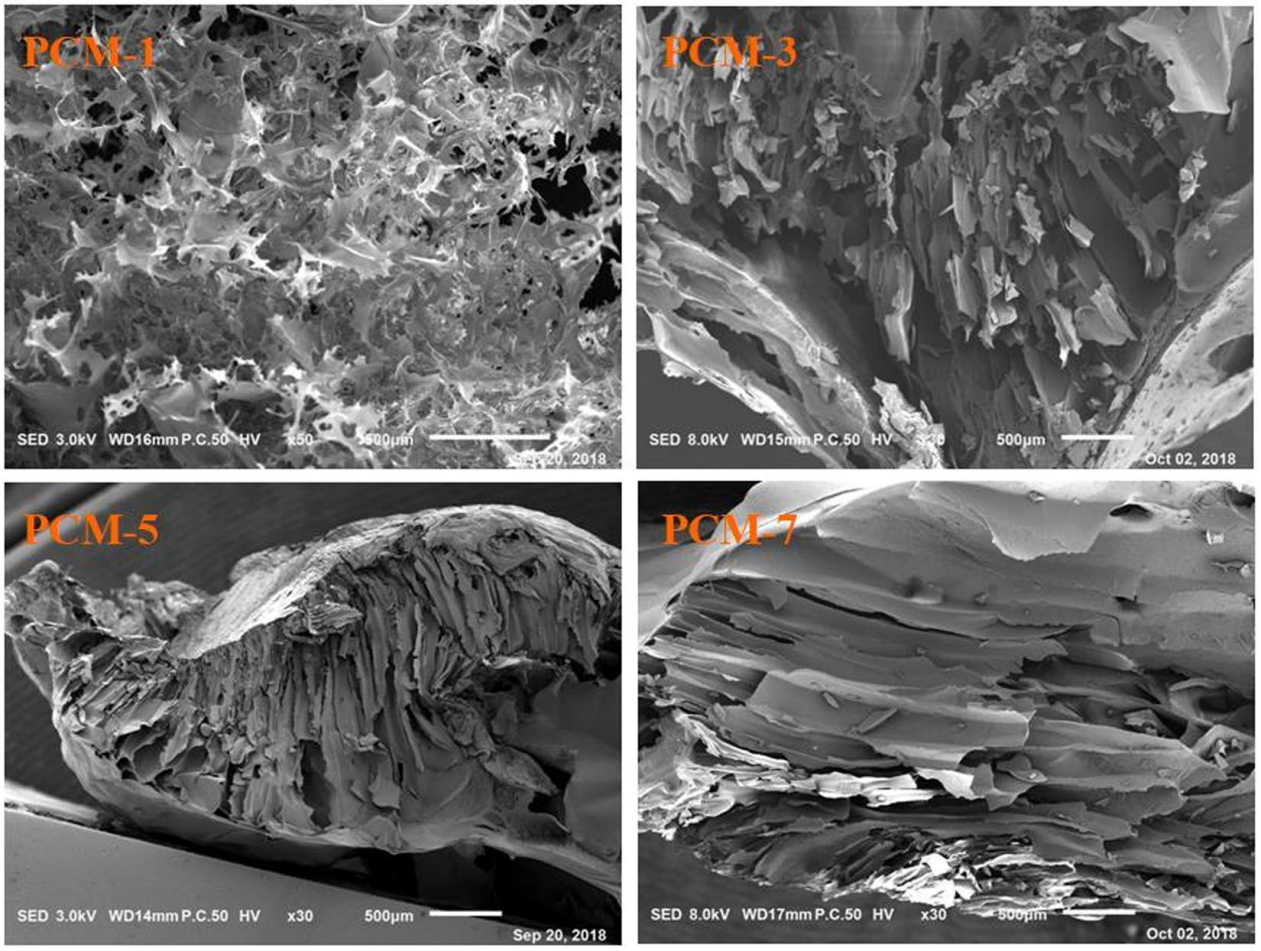
SEM images of the fracture surfaces for PCM-1, PCM-3, PCM-5, and PCM-7, respectively.

SEM images of the fracture surfaces of the porous cellulose materials PCM-1, PCM-3, PCM-5, and PCM-7 are shown in Fig. 3. PCM-1 has a fluffy and porous structure which is composed of randomly oriented cellulose sheets, with the sheets being twisted and broken. Different from PCM-1, PCM-3, PCM-5, and PCM-7 exhibit long channel structures which were composed of adjacent sheets. This is an indication that the concentration of cellulose solution significantly affects the morphology of the cellulose material.

It is interesting to find that the morphologic structures of the cellulose materials prepared from 3–5% cellulose solution are quite similar to that reported by Xu *et al*. in which the porous cellulose material was prepared by dissolving 5% of cellulose in neat [A_2_im][CH_3_OCH_2_COO]^20^. This reveals that the cellulose solution concentration and [A_2_im][CH_3_OCH_2_COO] dominate the morphologic structures of the cellulose material.

**Figure 4:**
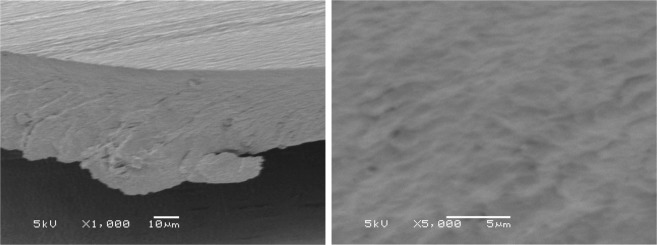
SEM images of the fracture surfaces for the regenerated cellulose films from [A_2_im][CH_3_OCH_2_COO]/NMP/cellulose solution at × 1000 and 5000 magnifications.

As a comparison, we also prepared a cellulose film, and its SEM images are shown in Fig. 4. As shown in Fig. 4, the fracture surface of the film exhibits a homogeneous and dense structure. The morphology of the cellulose film is quite similar to those reported in the literatures^42,43^.

**Figure 5:**
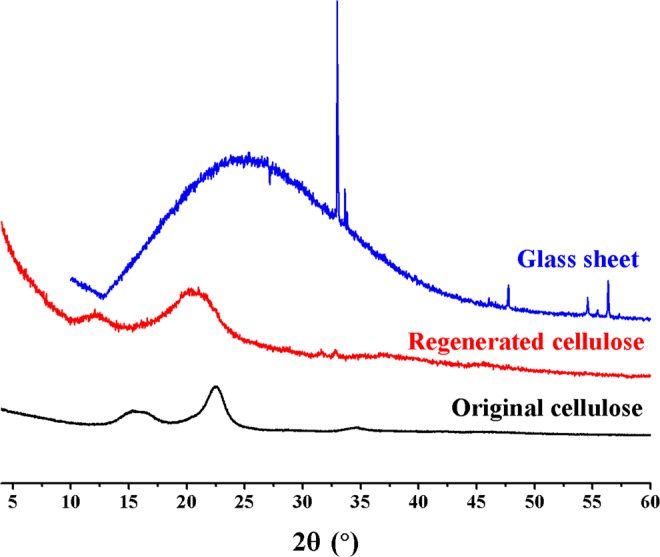
XRD spectra of the original cellulose and the regenerated cellulose from [A_2_im][CH_3_OCH_2_COO]/NMP/cellulose solution. Glass sheet was used to fix cellulose film.

Figure 5 shows the XRD patterns of the original cellulose and regenerated cellulose film. It is indicated in Fig. 5 that the original cellulose displays the typical diffraction peaks of cellulose I at 2θ = 15.2°, 16.4°, 22.5°, 34.6°^44^. In the meantime, the typical diffraction patterns of cellulose II are observed at 2θ = 12.5°, 20.3° and 21.2° for the regenerated cellulose exhibits^45^. This is indicative of a conversion from cellulose I to cellulose II.

**Figure 6:**
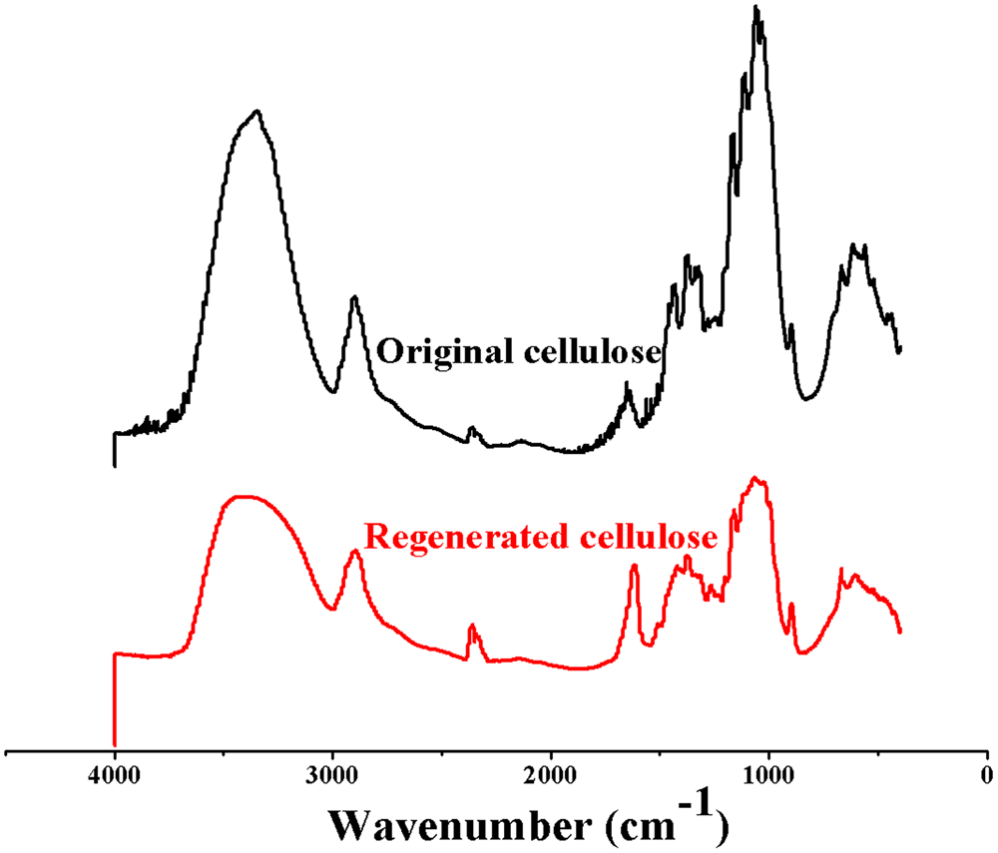
FT-IR spectra of the original cellulose and the cellulose regenerated from [A_2_im][CH_3_OCH_2_COO]/NMP/cellulose solution.

Figure 6 shows the FTIR spectra of the original and the regenerated cellulose. The FTIR spectra of the regenerated cellulose from [A_2_im][CH_3_OCH_2_COO]/NMP/cellulose solution are in excellent line with those of the original cellulose, thus implying that cellulose does not react with the [A_2_im][CH_3_OCH_2_COO]/NMP solvent in the dissolution and regeneration processes. The detailed affiliation for the absorption bands of the original and regenerated cellulose are placed in Supplementary Information. The FTIR spectra of the original and regenerated cellulose are similar to those reported in the literatures^43,46–49^.

## Conclusions

This work presents novel cellulose solvents [A_2_im][CH_3_OCH_2_COO]/NMP by mixing NMP with [A_2_im][CH_3_OCH_2_COO]. Attractively, the solvents display much efficient dissolution capacity for cellulose and gain as high as 25.4 g 100 g^−1^ of cellulose solubility even at 25 °C. The efficient cellulose dissolution is primarily ascribed to the hydrogen bond interactions of the carboxyl oxygen atom in [CH_3_OCH_2_COO]^−^ with the hydroxyl hydrogen in cellulose as well as the acidic protons in [A_2_im]^+^ with the hydroxyl oxygen. The role of NMP mainly dissociates [A_2_im][CH_3_OCH_2_COO] into [A_2_im]^+^ and [CH_3_OCH_2_COO]^−^ and stabilize the dissolved cellulose chains. The systematic analysis verifies that the morphology of the cellulose material mainly depends on the preparation approach, [A_2_im][CH_3_OCH_2_COO] and cellulose solution concentration. FT-IR analysis reveals that cellulose does not react with the [A_2_im][CH_3_OCH_2_COO]/NMP solvent in the dissolution and regeneration processes. XRD studies confirm the conversion from cellulose I to cellulose II after the original cellulose is dissolved and regenerated in [A_2_im][CH_3_OCH_2_COO]/NMP solvent. Therefore, this work provides a viable strategy for the practical application in cellulose processing/conversion even at as low as 25 °C.

## Supplementary information

Cellulose dissolution in diallylimidazolium methoxyacetate + N-methylpyrrolidinone mixture
