# Supplementary material for: Cellulose dissolution in diallylimidazolium methoxyacetate + N-methylpyrrolidinone mixture
Source: Sci Rep. 2019 Aug 8;9:11518. doi: 10.1038/s41598-019-48066-8 (PMC6687689; doi:10.1038/s41598-019-48066-8)
Supplement: Supplementary file 1 — Cellulose dissolution in diallylimidazolium methoxyacetate + N-methylpyrrolidinone mixture [file 41598_2019_48066_MOESM1_ESM.doc]

**Supporting Information**

**Cellulose dissolution in diallylimidazolium methoxyacetate + *N*-methylpyrrolidinone mixture**

**Airong Xu^a^,*,** **Yongxin Wang^a^,** **Rukuan Liu ^b^,***

^a^ School of Chemical Engineering and Pharmaceutics, Henan University of Science and Technology, Luoyang, Henan 471003, PR China

^b^ *Hunan Academy of Forestry, Changsha, Hunan 410004, PR China*


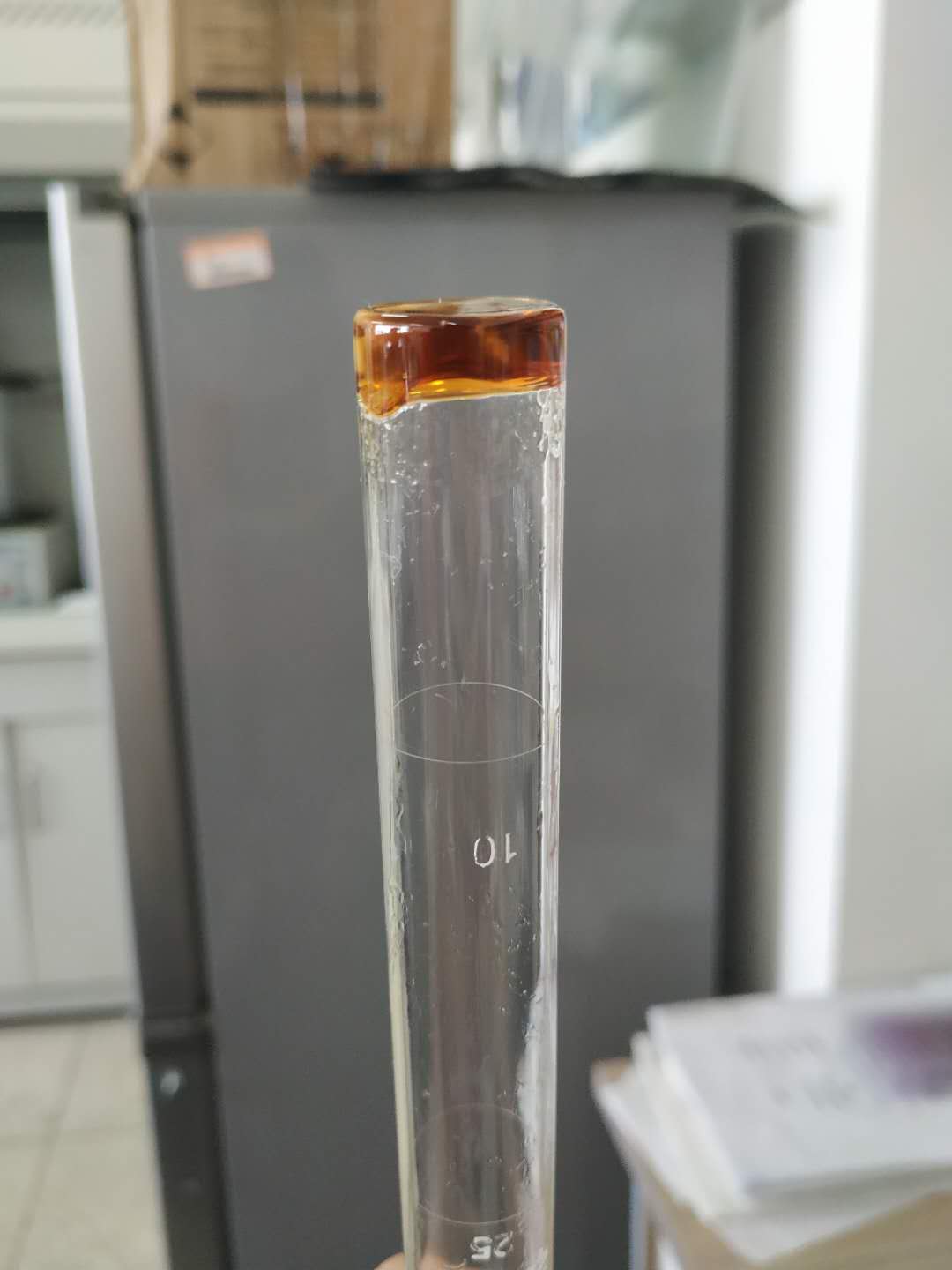


**Fig. S1** Photo of 5.2 % of [A_2_im][CH_3_OCH_2_COO]/NMP(*R*_NMP_ =2.43)/absorbent cotton solution at 50 °C.

**Fig. S2** ^13^C NMR spectra of [A_2_im][CH_3_OCH_2_COO] in [A_2_im][CH_3_OCH_2_COO]/NMP(*R*_NMP_=2) solvent at room temperature.

**Fig. S3** ^13^C NMR spectra of [A_2_im][CH_3_OCH_2_COO] in [A_2_im][CH_3_OCH_2_COO]/NMP(*R*_NMP_=2)/cellulose(8%) solution at room temperature.

**FTIR spectra analysis of the original** **and the regenerated cellulose**

The absorption band at 1423 cm^-1^ in the regenerated cellulose film is assigned to the CH_2_ scissoring vibration. This band was weakened and shifted to a lower wavenumber compared to the peak at 1431 cm^-1^ for the original cellulose, suggesting the destruction of an intra-molecular hydrogen bond involving O6. A new shoulder at 990 cm^-1^ in the regenerated cellulose film could be assigned to the C-O stretching vibration in the amorphous region. The O-H vibration in the regenerated cellulose film shifts to a higher wavenumber (3419 cm^-1^), which is a hint for the breaking of hydrogen bonds to some extent. The absorption bands in the range of 1164-1061 cm^-1^ belong to the C-O-C stretching of the original cellulose. The presence of such bands in the absorption of the regenerated cellulose suggests that the macromolecular structure of cellulose is not destructed after regeneration of the cellulose.
